# Effect of RUNX1/FOXP3 axis on apoptosis of T and B lymphocytes and immunosuppression in sepsis

**DOI:** 10.1515/med-2023-0728

**Published:** 2023-08-24

**Authors:** Yangfa Chao, Wenting Huang, Zhiheng Xu, Ping Li, Shaodong Gu

**Affiliations:** Department of Surgical Area 4, Shenzhen Bao’an Traditional Chinese Medicine Hospital Group, Shenzhen, Guangdong Province, 518000, China; Department of Acupuncture, Luohu District Chronic Disease Prevention and Treatment Hospital, Shenzhen, China; The Second Department of Surgery, The First Affiliated Hospital of Guangzhou University of Traditional Chinese Medicine, Guangzhou, China; Department of Surgical Area 4, Shenzhen Bao’an Traditional Chinese Medicine Hospital Group, No. 25 Yu’an 2nd Road, Bao’an District, Shenzhen, Guangdong Province, 518000, China

**Keywords:** sepsis, lymphocytes, apoptosis, RUNX1, FOXP3

## Abstract

Lymphocyte apoptosis is a latent factor for immunosuppression in sepsis. Forkhead box protein P3 (FOXP3) can interact with RUNX family transcription factor 1 (RUNX1) in regulatory T cells. Our research was to probe whether RUNX1/FOXP3 axis affects immunosuppression in the process of sepsis by modulating T and B lymphocyte apoptosis. We constructed sepsis model in mice and mouse CD4^+^ T and CD19^+^ B lymphocytes. RUNX1 and FOXP3 expressions and apoptosis in cells were assessed by western blot, quantitative real-time PCR, and flow cytometer. Inflammation of serum and pathological damage was assessed by ELISA and H&E staining. Relationship between RUNX1 and FOXP3 was assessed by co-immunoprecipitation. The findings showed that RUNX1 ameliorated the survival rate, pathological damage, and decreased inflammation-related factors, and inhibited apoptosis of CD4^+^ T and CD19^+^ B cells in cecal ligation and puncture mice. Furthermore, RUNX1 up-regulated the viability and down-regulated apoptotic rate with the changed expressions of apoptosis-related molecules in lipopolysaccharide (LPS)-mediated CD4^+^ T and CD19^+^ B cells. Additionally, FOXP3 interacted with RUNX1, and its silencing decreased RUNX1 expression and reversed the inhibitory effect of RUNX1 on apoptosis of LPS-mediated CD4^+^ T and CD19^+^ B cells. In summary, the RUNX1/FOXP3 axis alleviated immunosuppression in sepsis progression by weakening T and B lymphocyte apoptosis.

## 1 Introduction

Sepsis is a fatal organ function damage caused by the uncontrolled immune response of the body to infection, and is a significant cause of death in critically ill patients [1]. Epidemiological studies manifested that there were six million deaths from sepsis worldwide every year, and the mortality of severe sepsis is as high as 25–30% [2]. Some studies on the prognosis of sepsis have pointed out that surviving sepsis patients have poor long-term survival, with physical and psychological dysfunction, decreased quality of life, increased risk of readmission, and become a burden on family and society [3,4].

In the early stage of sepsis, a large number of pro-inflammatory factors and oxygen free radicals are produced under the stimulation of pathogenic microorganisms and metabolites. With the continuous improvement of organ protection and alternative treatment technologies, patients with sepsis can pass through the period of excessive inflammatory response and enter the long-term immunosuppressive state [5]. Mounting studies have exhibited that immunosuppression is the main pathophysiological mechanism in the initiation and development of sepsis and severe immunosuppression is a considerable element leading to the deterioration of the patient’s condition [6]. It has been reported that lymphocyte apoptosis is tightly correlated with immunosuppression [7]. If lymphocyte apoptosis is a significant mechanism, then specific lymphocyte subsets may be more vulnerable [8].

RUNX family transcription factor 1 (RUNX1) is a gene related to immunosuppression, which can regulate the expression of a variety of transcription factors; the study unveiled that deletion of RUNX1 can cause autoimmune lung disease in mice [9]. Hsu et al. have reported that loss of RUNX1 function resulted in the loss of CD4^+^ T cells [10]. Some scholars have illustrated that knockdown of RUNX1 led to B cell immunodeficiency in adult zebrafish [11]. Another research has clarified that miR-338-3p mimic impedes Treg-induced immunosuppression in pemphigus vulgaris through modulating RUNX1 expression [12]. In addition, Zhang et al. has reported that exosomal miR-21-5p mitigated sepsis-triggered acute kidney damage through restraining RUNX1 level [13]. However, whether RUNX1 affects immunosuppression in the process of sepsis by modulating lymphocyte apoptosis remains to be further investigated. On the other hand, the Forkhead box protein P3 (FOXP3) is a master cell lineage regulator in CD4^+^ CD25^+^ natural regulatory T cell (Treg) development. FOXP3 can interact with RUNX1 in regulatory T cells [14,15]. Furthermore, FOXP3^+^ regulatory T cells are necessary for recovery from severe sepsis [16].

Based on the above research evidence, this study probes the role of RUNX1 on inflammation and pathological damage of liver, kidney, and lung tissues in cecal ligation and puncture (CLP) mice. Besides, we further explore the effect of RUNX1 on inflammation response in serum and apoptosis in CD4^+^ T and CD19^+^ B cells from CLP mice. Meanwhile, CD4^+^ T and CD19^+^ B cells induced by lipopolysaccharide (LPS) were served as the experimental subjects *in vitro* to analyze the role and mechanism of RUNX1 with FOXP3 in CD4^+^ T and CD19^+^ B cells.

## 2 Materials and methods

### 2.1 Animals

We bought 100 male specific pathogen-free C57BL/6 mice (8–12 week old) from Chengdu Dashuo Experimental Animal Co., Ltd (China). They were acclimatized for 7 days under the same facilities (22–24℃, 60 ± 2% humidity, 12 h light/dark cycle).

### 2.2 CLP model

CLP surgery was performed on 75 mice to construct a sepsis model [17]. To relieve pain, all mice received buprenorphine (0.1 mg/kg, B-044, Supelco, USA) before surgery. First, mice were anesthetized by intraperitoneal injection with 0.3% pentobarbital sodium (P3761, Sigma-Aldrich, USA). A 1 cm incision was made in the abdominal midline of the mice. Next, we ligated the cecum approximately 1 cm from the end of the cecum using a 4-0 surgical suture. Then, we used no. 21 needle to puncture the ligated cecum twice. Finally, the abdominal incision was sutured layer by layer. The remaining 25 mice belonged to the control group and underwent sham surgery. Mice in the control group were only treated with laparotomy and cecum explantation. All mice were injected subcutaneously with ceftriaxone (25 mg/kg, C304513, Aladdin, China) and metronidazole (12.5 mg/kg, M109873, Aladdin, China) after surgery and resuscitated with 1 mL of normal saline. To observe the survival of mice, we observed the animals every 12 hours within 10 days after operation. A total of 60 mice were used for survival study.

### 2.3 Animal treatment

The lentiviral vectors expressing the plasmids (RUNX1 or corresponding negative controls [empty vector]) were injected into mice with sepsis at a titer of 1  ×  10^9^ via the tail vein. Injection was conducted 48 h after CLP.

### 2.4 Isolation of CD4^+^ T and CD19^+^ B lymphocytes from mouse spleen

We first harvested the spleen tissues of C57BL/6 mice, and then prepared a single cell suspension, as previously reported [17]. Then, the red blood cells were lysed and primary mouse CD19^+^ B and CD4^+^ T lymphocytes were isolated using a MagniSort™ Mouse CD19 Positive Selection Kit (8802-6847-74, Invitrogen, USA) and Dynabeads™ Untouched™ Mouse CD4 Cells Kit (11416D, Invitrogen, USA), respectively. Primary CD4^+^ T and CD19^+^ B cells were kept in RPMI-1640 medium (22400089, Gibco, USA) augmented with 10% fetal bovine serum (10099141, Gibco, USA) and 1% penicillin–streptomycin liquid (P1400, Solarbio, China) at 37℃ with 5% CO_2_.

### 2.5 ELISA

Mice were euthanized after Day 5 of CLP, and then we collected whole blood through a cardiac puncture. The blood samples were centrifuged (1,000*g*, 10 min), and the supernatant was gained and stored at −80℃ until further analysis. Mouse tumor necrosis factor α (TNF-α) kit (BPE20220), interleukin 6 (IL-6) kit (BPE20012), and IL-1β kit were ordered from Lengton (China). Serum samples (50 μL) and biotin antigen working solution (50 μL) were added to the sample wells, and then reacted at room temperature (RT) for 0.5 h. After thoroughly cleaning each well, 50 μL avidin-horseradish peroxidase (HRP) was added to the sample wells and reacted at RT for 0.5 h. After fully washing each well again, chromogenic agents A and B were successively added to each well. After 10 min of reaction at RT, we added stop solution to each well. Lastly, we measured the absorbance (450 nm) of each well with the aid of a microplate reader (RT-6000; Rayto, China).

### 2.6 Apoptosis analysis in CD4^+^ T cells and CD19^+^ B cells from mice

The whole blood (100 μL) was lysed with VersaLyse lysing solution (A09777, Beckman-Coulter, Hialeah, FL, USA) for 15 min at RT. After washing, cells were incubated with phycoerythrin-labeled antibodies directed against CD4 and CD19 (CD4^+^ T cells and CD19^+^ B cells). RIPA buffer (R0010, Solarbio, China) was taken to lyse the cells. After rinsing, the lysed samples were subjected to PE Annexin V Apoptosis Detection Kit (559763, BD Biosciences, USA) based on kit instructions. Then, the results were assessed with the help of a flow cytometer (PAS III, Partec, Germany) within 0.5 h. Regarding the caspase-3 intracellular staining, fixation/permeabilization solution kit (554715, BD Biosciences, USA) was taken to fix and permeate the cells. Subsequently, PE rabbit anti-active caspase-3 antibody (561011, BD Biosciences, USA) was added. After cells were rinsed, we utilized the flow cytometer to estimate the results. Data are presented as percentages of respective Annexin V^+^ or caspase-3^+^ cell populations. A positive threshold was established based on non-stained control cells.

### 2.7 H&E staining

H&E staining kit (G1120, Solarbio, China) was utilized in this research. We harvested the liver tissues, kidney tissues, and lung tissues from the mice. All tissues were fixed in 4% paraformaldehyde (G1101, Servicebio, China). The fixed tissues were dehydrated, embedded in paraffin, and cut into 3 μm slices. Next, the slices were deparaffinized in xylene, rehydrated in ethanol, and then stained utilizing hematoxylin solution. After that, the slices were subjected to differentiation. After being stained with eosin solution, the slices went through ethanol dehydration and xylene transparent. Lastly, the samples were observed with the aid of an optical microscope (×40, ×100, Ts2R-FL) after being sealed through neutral balsam (D054-1-1, Jiancheng, China).

### 2.8 Cell transfection and grouping

T lymphocytes (CD4^+^ T and CD19^+^ B) were transfected with lentiviral vectors expressing shRNA against FOXP3 (sh-FOXP3), RUNX1 overexpression, or corresponding negative controls (empty vector). To probe the role of RUNX1 on cells, cells were assigned to the control group, LPS group (cells were subjected to 1 μg/mL LPS for 24 h), and LPS + vector/LPS + RUNX1 group (cells transfected with overexpressed RUNX1 or vector were exposed to 1 μg/mL LPS for 24 h). Next, to analyze the functions of RUNX1 and FOXP3 on cells, cells were separated into the LPS + vector group, LPS + RUNX1 group, LPS + sh-FOXP3 group, and LPS + RUNX1 + sh-FOXP3 group.

### 2.9 Cell viability analysis

CD4^+^ T and CD19^+^ B cells (1 × 10^4^ per well) plated into the 96-well plates were subjected to transfection and LPS intervention. Thereafter, a further 4 h culture was done after 10 μL MTT solution (M1020, Solarbio, China) was added. Then, the OD value (490 nm) was evaluated with the aid of the microplate reader after being added to 110 μL formazan solution.

### 2.10 Apoptosis experiment

Apoptosis of CD4^+^ T and CD19^+^ B cells (1.2 × 10^6^) was estimated with the help of the Annexin V-FITC/PI kit (556547, BD, USA). The treated cells of each group were rinsed, digested, and then centrifuged. Cell precipitation was re-suspended in 1× binding buffer and cell concentration was adjusted to 1 × 10^6^ cells/mL. After that, 5 μL Annexin V-FITC and 10 μL PI were injected into each tube. After being reacted at 37℃ without light for 15 min, we utilized the flow cytometer to evaluate apoptosis of each group.

### 2.11 Co-immunoprecipitation (Co-IP)

Beyotime (China) provided the IP buffer (P0013). We collected CD4^+^ T and CD19^+^ B cells (1 × 10^5^ cells/mL). After being lysed with IP buffer, cells were subjected to ultrasonic treatment followed by centrifugation. The supernatant was then reacted with anti-rabbit IgG antibody (1:8,000, ab205718; Abcam, UK), anti-FOXP3 antibody (1:30, ab215206; Abcam, UK), anti-RUNX1 antibody (1:20, ab92336; Abcam, UK), and BeyoMag™ Protein A + G Magnetic Beads (P2173, Beyotime, China) at 4℃ overnight. Afterward, the complex was rinsed and the binding proteins were eluted with SDS sample buffer. In the end, the precipitated proteins were determined through western blot.

### 2.12 Western blot

CD4^+^ T cells and CD19^+^ B cells from mice were lysed through the RIPA buffer (WB038, GEFAN, China) [18]. The extracted protein was centrifuged and then quantified with the help of the BCA kit (23225, Thermo Scientific, USA). After being electrophoresed, they were transferred to the PVDF membrane before being blocked with 5% skim milk. Next, we utilized primary antibodies to treat the membrane at 4℃ all night. After that, second antibody was used to treat the membrane at 37℃ for 90 min. After rinsing, the reactive bands were reacted with the color reagent (34075, PIERCE, USA) in a gel imaging system (610020-9Q, Clinx, China). The primary antibodies of RUNX1 (1:1,000, ab240639, 48 kDa), FOXP3 (1:1,000, ab20034, 50 kDa), Bax (1:8,000, ab32503, 21 kDa), Bcl-2 (1:2,000, ab196495, 26 kDa), cleaved caspase-3 (1:5,000, ab214430, 17 kDa), caspase-3 (1:2,000, ab184787, 32 kDa), and GAPDH (1:10,000, ab181602, 36 kDa), and the second antibodies of goat anti-rabbit IgG H&L (HRP) (1:2,000, ab205718) and goat anti-mouse IgG H&L (HRP) (1:2,000, ab205719) were gained from Abcam (UK). GAPDH was exploited to the housekeeping gene. The protein level was normalized to GAPDH, and presented relative to control cells (set as 1).

### 2.13 Quantitative real-time PCR (qRT-PCR)

We obtained total RNA from cells with the help of the RNA extraction kit (HYY0420-50, Huayueyang, China). Subsequently, the one-step RT-qPCR system (A6020) offered by Promega (USA) was applied to conduct the qRT-PCR reaction. The reaction system was done with the aid of a PCR system (ABI7900, Applied Biosystems, USA). GAPDH was the housekeeping gene and the 2^−ΔΔCt^ was utilized for data evaluation [19]. Primer sequences are listed in Table 1.

**Table 1:** Primer sequences used in qRT-PCR

Genes	Forward (5′ → 3′)	Reverse (5′ → 3′)
RUNX1	ACGATGAAAACTACTCGGCAG	CTGAGGTCGTTGAATCTCGCT
FOXP3	CCCATCCCCAGGAGTCTTG	ACCATGACTAGGGGCACTGTA
GAPDH	TGACCTCAACTACATGGTCTACA	CTTCCCATTCTCGGCCTTG

### 2.14 Statistics

There were at least three independent experiments for data analysis. GraphPad Prism 8.0 (GraphPad Software Inc., USA) was exploited to process data. Quantitative data were manifested as mean ± standard deviation. Comparisons between two groups were assessed by an independent sample *t*-test. Comparisons among multiple groups were conducted utilizing a one-way ANOVA followed by Tukey’s *post hoc* test. A *P*-value < 0.05 was considered significant.


**Ethics statement:** All animal studies were done in compliance with the guidelines for China Animal Care and Use Committee. All animal operations were performed in Nanfang Hospital and were allowed by the Ethics Committee of Nanfang Hospital (2020041569). Every effort was made to minimize pain and discomfort to the animals.

## 3 Results

### 3.1 Down-regulation of RUNX1 in CD4^+^ T and CD19^+^ B cells from CLP mice was reversed by overexpressed RUNX1, and FOXP3 was lowly expressed in CD4^+^ T and CD19^+^ B cells

**Figure 1:**
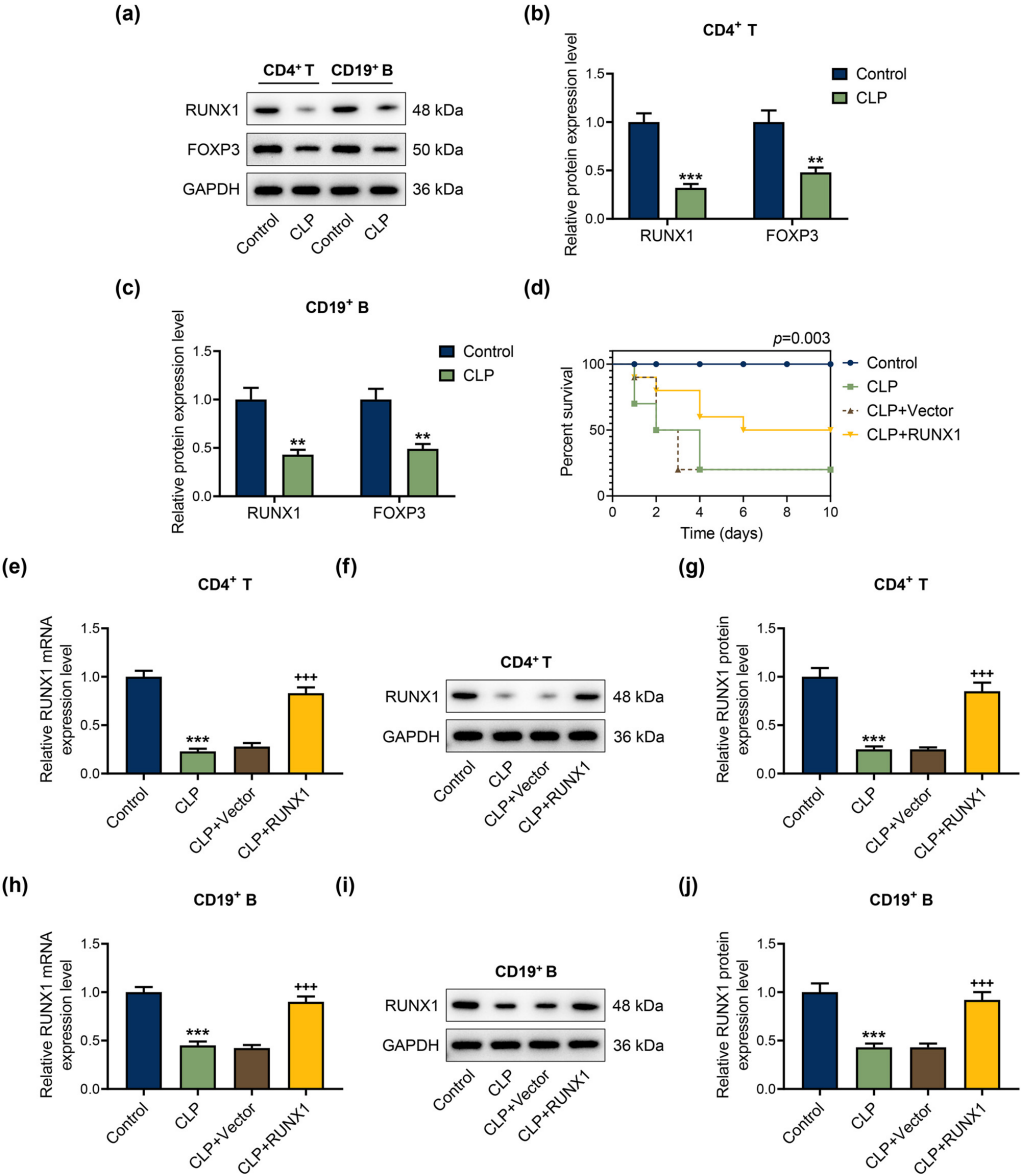
Down-regulation of RUNX1 in CD4^+^ T and CD19^+^ B cells from CLP mice was reversed by overexpressed RUNX1, and FOXP3 was lowly expressed in CD4^+^ T and CD19^+^ B cells (a)–(c) RUNX1 and FOXP3 expressions in CD4^+^ T and CD19^+^ B cells of mice were assessed by western blot. (d) Survival rate of mice in each group. (e)–(j) Role of overexpressed RUNX1 on RUNX1 expression in CD4^+^ T and CD19^+^ B cells of CLP mice was examined by qRT-PCR and western blot, with GAPDH as the endogenous gene. ^**^ *P* < 0.01, ^***^ *P* < 0.001 vs control; ^+++^ *P* < 0.001 vs CLP + Vector.

The expressions of RUNX1 and FOXP3 in CD4^+^ T and CD19^+^ B cells of CLP mice were lower than those in the control group (Figure 1a–c, *P* < 0.01). Next, we assessed the survival rate of mice in each group. The survival rate of CLP mice was lower than that of the control group, while RUNX1 overexpression ameliorated the survival rate of CLP mice (Figure 1d, *P* = 0.003). After injecting RUNX1 overexpression vectors, the mRNA and protein levels of RUNX1 in CD4^+^ T and CD19^+^ B cells from CLP mice were notably augmented (Figure 1e–j; *P* < 0.05). The data indicated that RUNX1 and FOXP3 was lowly expressed in CD4^+^ T and CD19^+^ B cells, and RUNX1 overexpression vectors reversed the down-regulation of RUNX1 in CD4^+^ T and CD19^+^ B cells from CLP mice.

### 3.2 Up-regulation of inflammatory factor in serum and apoptosis in CD4^+^ T and CD19^+^ B cells from CLP mice was partially offset by RUNX1 overexpression

**Figure 2:**
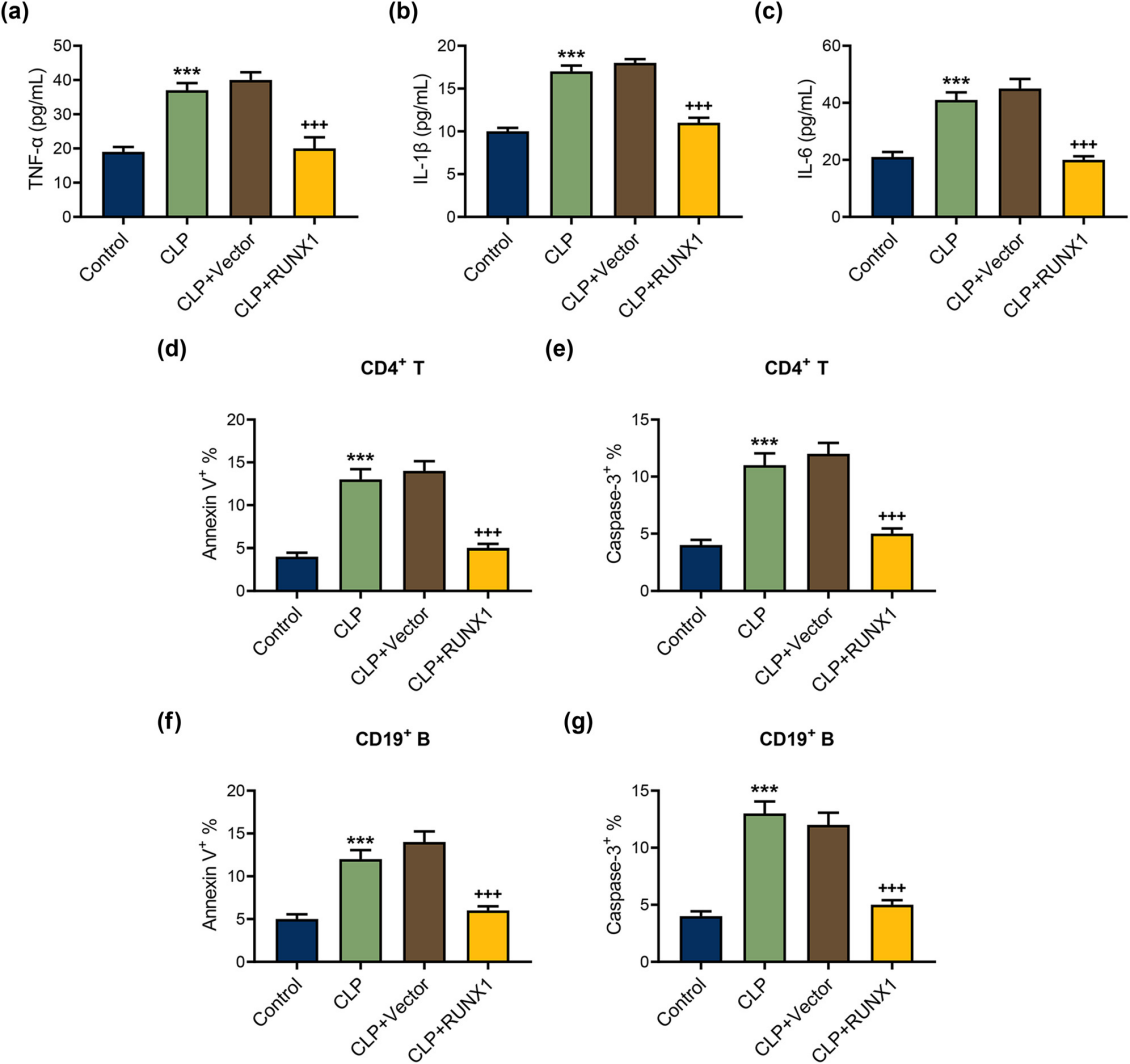
Up-regulation of inflammatory factor and apoptosis in CD4^+^ T and CD19^+^ B cells from CLP mice was partially offset by RUNX1 overexpression. The role of overexpressed RUNX1 on inflammatory factor (a)–(c) and apoptosis (d)–(g) in CD4^+^ T and CD19^+^ B cells from CLP mice was examined by ELISA and flow cytometer. ^***^ *P* < 0.001 vs control; ^+++^ *P* < 0.001 vs CLP + Vector.

Then, we utilized ELISA to determine the content of inflammatory factors in the serum of mice in each group. TNF-α, IL-1β, and IL-6 contents in the serum of mice in the CLP group were higher than the control group, whereas RUNX1 overexpression evidently attenuated TNF-α, IL-1β, and IL-6 contents in the serum of mice (Figure 2a–c, *P* < 0.001). We further discovered a notable elevation of annexin-V binding and active caspase-3 in CD4^+^ T and CD19^+^ B cells of CLP mice (Figure 2d–g, *P* < 0.001). Importantly, overexpressed RUNX1 repressed annexin-V binding and active caspase-3 in CD4^+^ T and CD19^+^ B cells of CLP mice (Figure 2d–g, *P* < 0.001). The data indicated that RUNX1 overexpression exerts inhibitory effects on the content of inflammatory factors in serum and apoptosis in CD4^+^ T and CD19^+^ B cells from CLP mice.

### 3.3 RUNX1 overexpression alleviated liver, kidney, and lung injury in CLP mice

**Figure 3:**
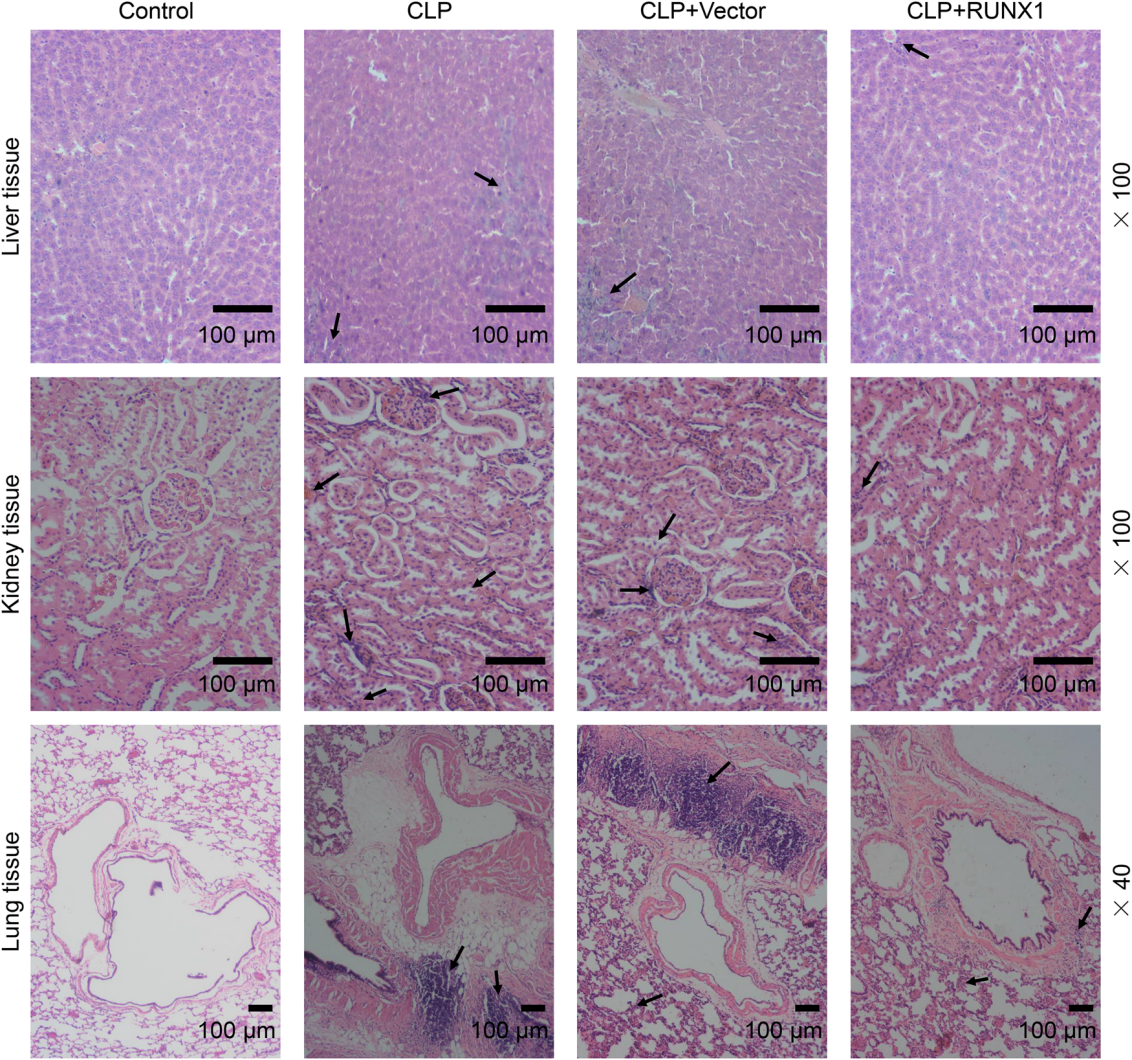
RUNX1 overexpression alleviated liver, kidney, and lung injury in CLP mice. The effect of overexpressed RUNX1 on liver, kidney, and lung injury in CLP mice was examined by H&E staining (100×, 40×, 100 μm). Infiltration of inflammatory cells and the damaged sites are indicated by arrows.

In order to probe the organ damage of mice in each group, we harvested the liver, kidney, and lung tissues of mice for H&E staining. Compared with the control group, CLP mice had increased alveolar wall thickness and inflammatory cell infiltration; CLP also resulted in evident necrosis of liver tissue and exfoliation of renal tubular epithelial cells in mice (Figure 3). Application of RUNX1 overexpression ameliorated liver, kidney, and lung injury in CLP mice (Figure 3). The data indicated that RUNX1 overexpression alleviated liver, kidney, and lung injury in CLP mice.

### 3.4 RUNX1 up-regulation intensified RUNX1 expression and cell viability in LPS-mediated CD4^+^ T and CD19^+^ B cells

**Figure 4:**
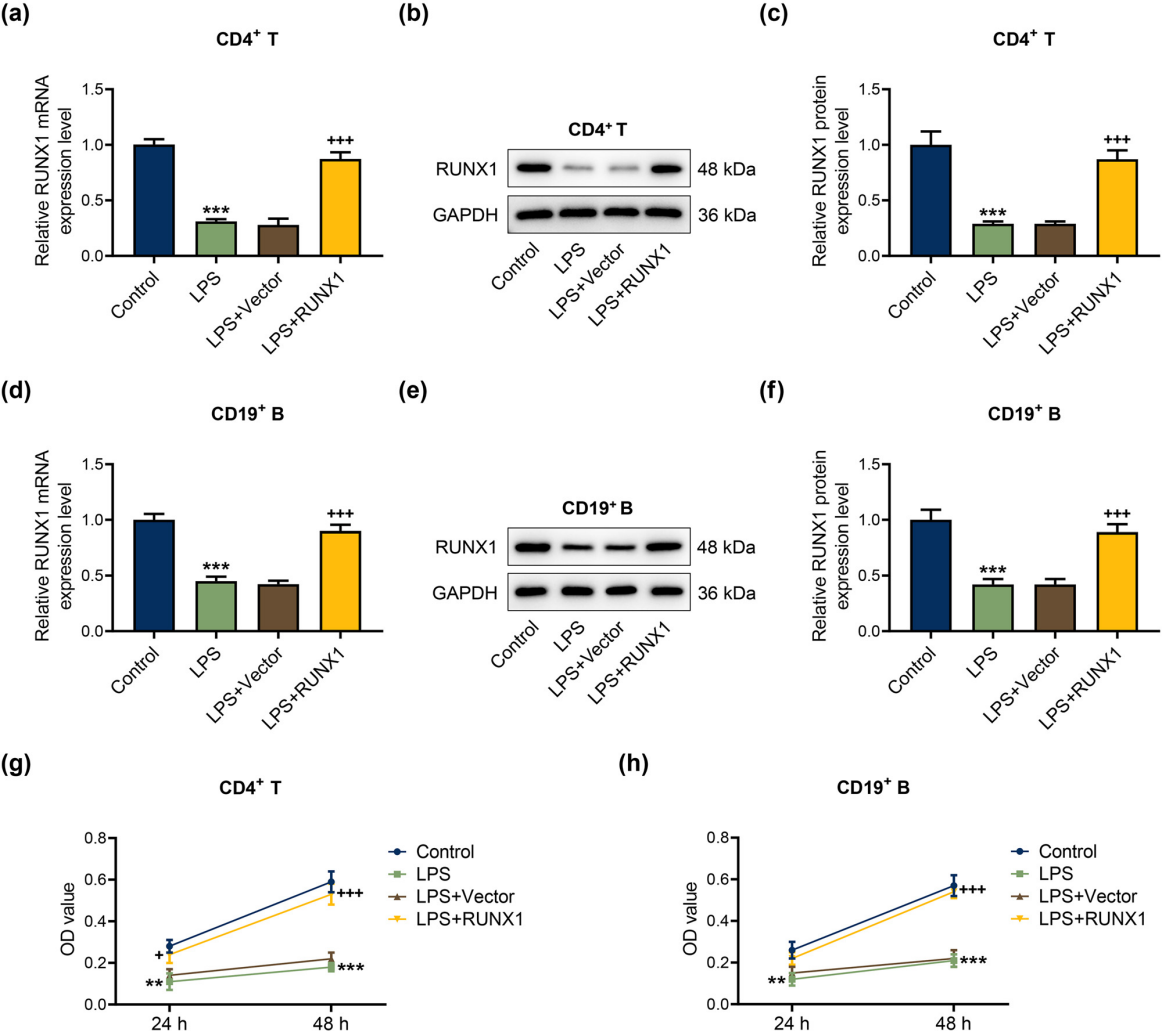
RUNX1 up-regulation intensified RUNX1 expression and cell viability in LPS-mediated CD4^+^ T and CD19^+^ B cells. (a)–(f) Effect of RUNX1 up-regulation on the mRNA and protein expressions of RUNX1 was assessed by qRT-PCR and western blot, with GAPDH as the endogenous gene. (g) and (h) Effect of RUNX1 up-regulation on cell viability in LPS-mediated CD4^+^ T and CD19^+^ B cells was estimated by MTT. ^**^ *P* < 0.01, ^***^ *P* < 0.001 vs control; ^+^ *P* < 0.05, ^+++^ *P* < 0.001 vs LPS + Vector.

To probe the role of RUNX1 on CD4^+^ T and CD19^+^ B cells, cells transfected with or without RUNX1 overexpression vector were reacted with 1 μg/mL LPS for 24 h. As displayed in Figure 4a–f, LPS caused the down-regulation of the mRNA and protein levels of RUNX1, while overexpressed RUNX1 reversed its effects (*P* < 0.01). Meanwhile, overexpressed RUNX1 largely elevated the cell viability of CD4^+^ T and CD19^+^ B cells induced by LPS (Figure 4g and h, *P* < 0.05). The data indicated that RUNX1 overexpression elevated the expression of RUNX1 and cell viability of CD4^+^ T and CD19^+^ B cells during LPS condition.

### 3.5 RUNX1 up-regulation regulated apoptosis rate, the expressions of apoptosis-related markers, and FOXP3 expressions in CD4^+^ T and CD19^+^ B cells induced by LPS

**Figure 5:**
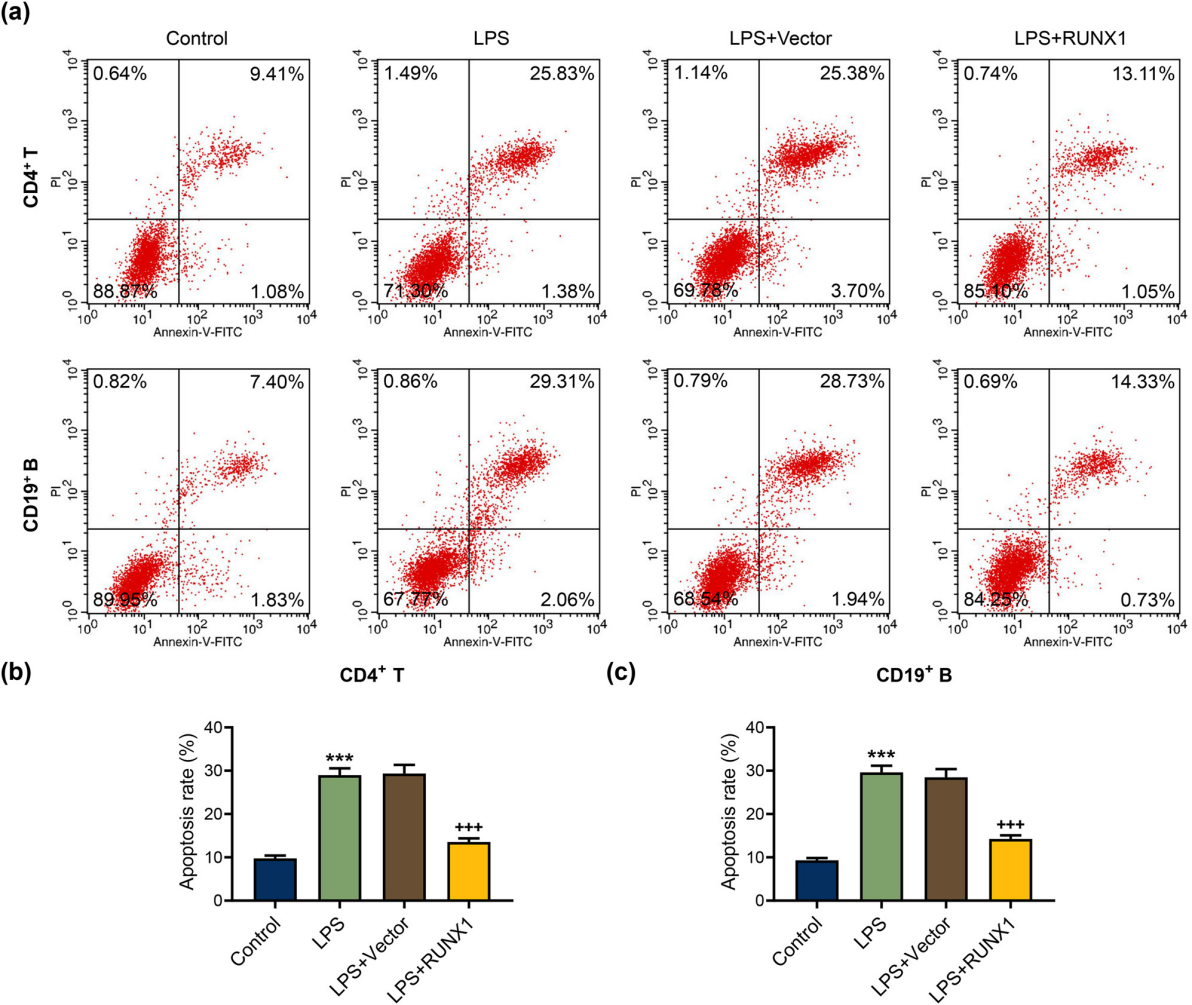
RUNX1 up-regulation repressed apoptosis rate in CD4^+^ T and CD19^+^ B cells induced by LPS. (a)–(c) The effect of RUNX1 up-regulation on apoptosis rate in CD4^+^ T and CD19^+^ B cells induced by LPS was assessed by flow cytometer. ^***^ *P* < 0.001 vs control; ^+++^ *P* < 0.001 vs LPS + Vector.

**Figure 6:**
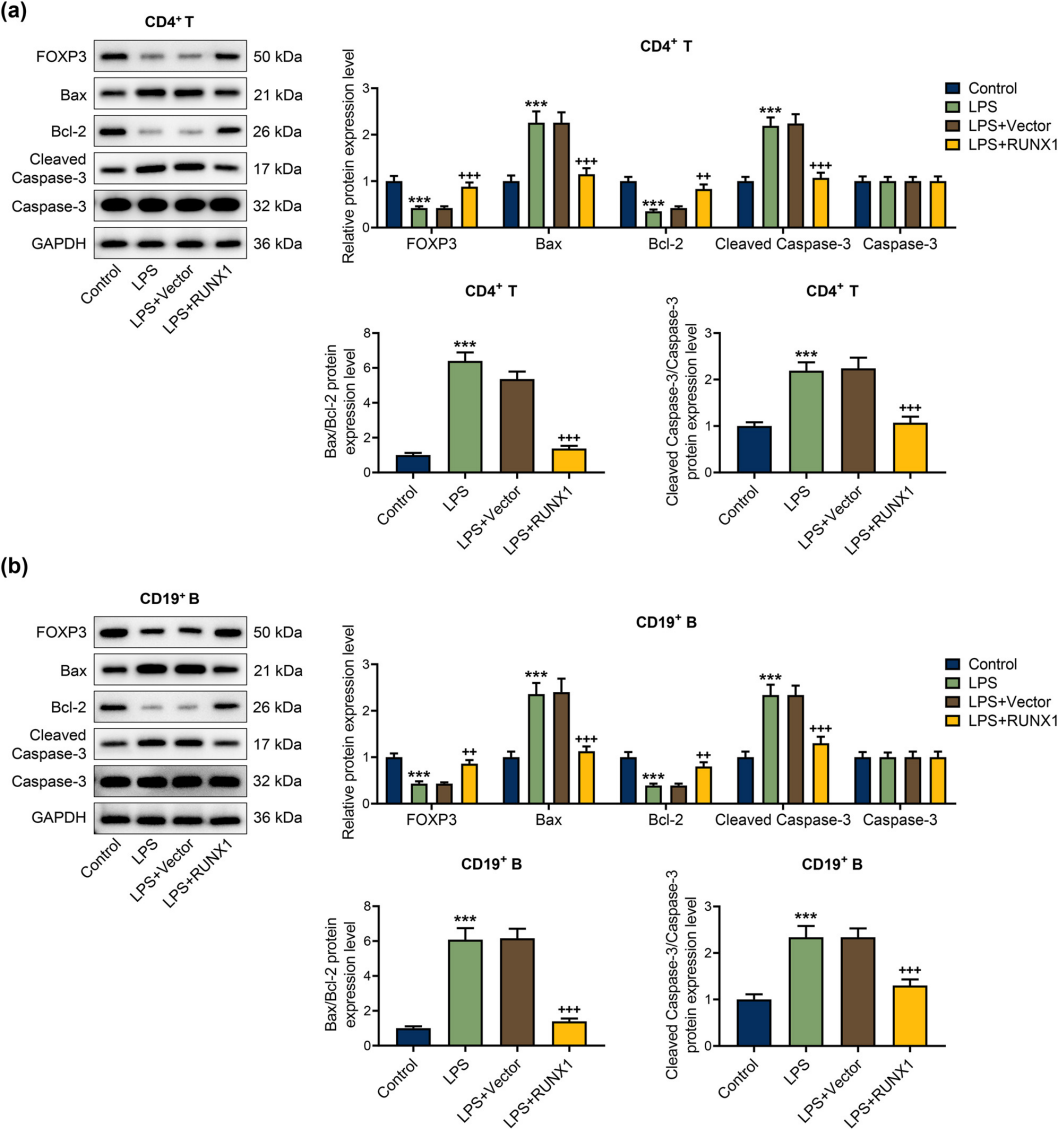
RUNX1 up-regulation regulated apoptosis-related markers and FOXP3 expressions in CD4^+^ T and CD19^+^ B cells induced by LPS. (a) and (b) Effect of RUNX1 up-regulation on FOXP3 expression and apoptosis-related marker expressions in CD4^+^ T and CD19^+^ B cells induced by LPS was assessed by western blot. ^***^ *P* < 0.001 vs control; ^++^ *P* < 0.01, ^+++^ *P* < 0.001 vs LPS + Vector.

The flow cytometer analysis exhibited that RUNX1 up-regulation extremely mitigated apoptosis rate of CD4^+^ T and CD19^+^ B cells triggered by LPS (Figure 5a–c, *P* < 0.001). At the molecular level, we discovered that LPS resulted in the reduction of FOXP3 and Bcl-2 and the increase of Bax, cleaved caspase-3, the ratio of cleaved caspase-3/caspase-3, and Bax/Bcl-2, whereas RUNX1 up-regulation partially offset the effect of LPS (Figure 6a and b, *P* < 0.001). The data indicated that RUNX1 overexpression repressed apoptosis and elevated FOXP3 expression in CD4^+^ T and CD19^+^ B cells under LPS condition.

### 3.6 FOXP3 interacted with RUNX1 in CD4^+^ T and CD19^+^ B cells

**Figure 7:**
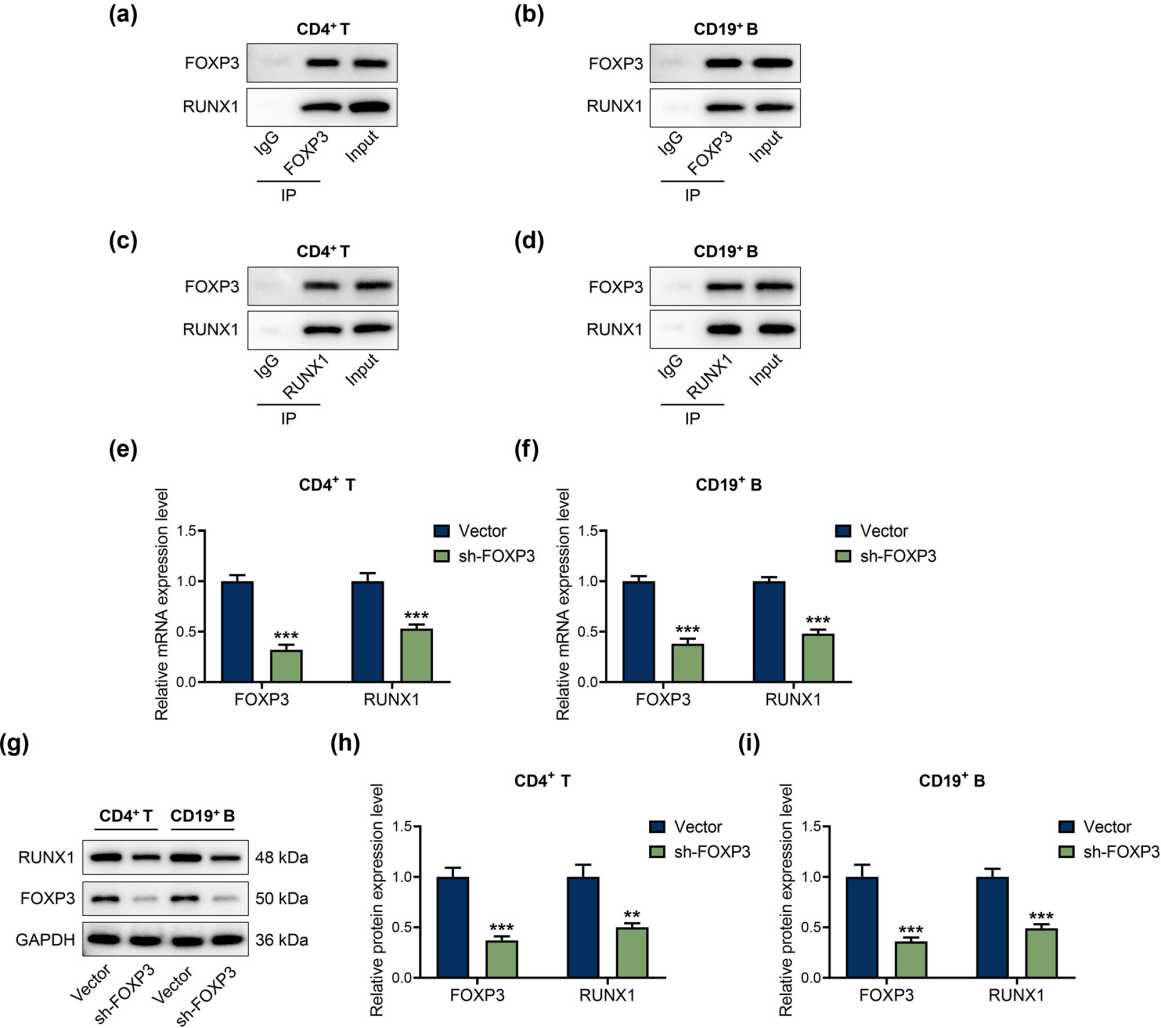
FOXP3 interacted with RUNX1. (a)–(d) Relationship between RUNX1 and FOXP3 was assessed by Co-IP. (e)–(i) RUNX1 and FOXP3 expressions in CD4^+^ T and CD19^+^ B cells transfected with sh-FOXP3 were assessed by qRT-PCR and western blot, with GAPDH as the endogenous gene. ^**^ *P* < 0.01, ^***^ *P* < 0.001 vs Vector.

In the next research, we examined the relationship between RUNX1 and FOXP3 with the help of CO-IP. As exhibited in Figure 7a–d, the results clarified that RUNX1 and FOXP3 formed a complex and interacted with each other in the CD4^+^ T and CD19^+^ B cells. Moreover, we also uncovered that FOXP3 silencing greatly weakened the mRNA and protein expressions of RUNX1 and FOXP3 in CD4^+^ T and CD19^+^ B cells (Figure 7e–i, *P* < 0.01). The data indicated that FOXP3 interacted with RUNX1 in CD4^+^ T and CD19^+^ B cells.

### 3.7 FOXP3 silencing reversed the suppression of overexpressed RUNX1 on apoptosis in LPS-mediated CD4^+^ T and CD19^+^ B cells

**Figure 8:**
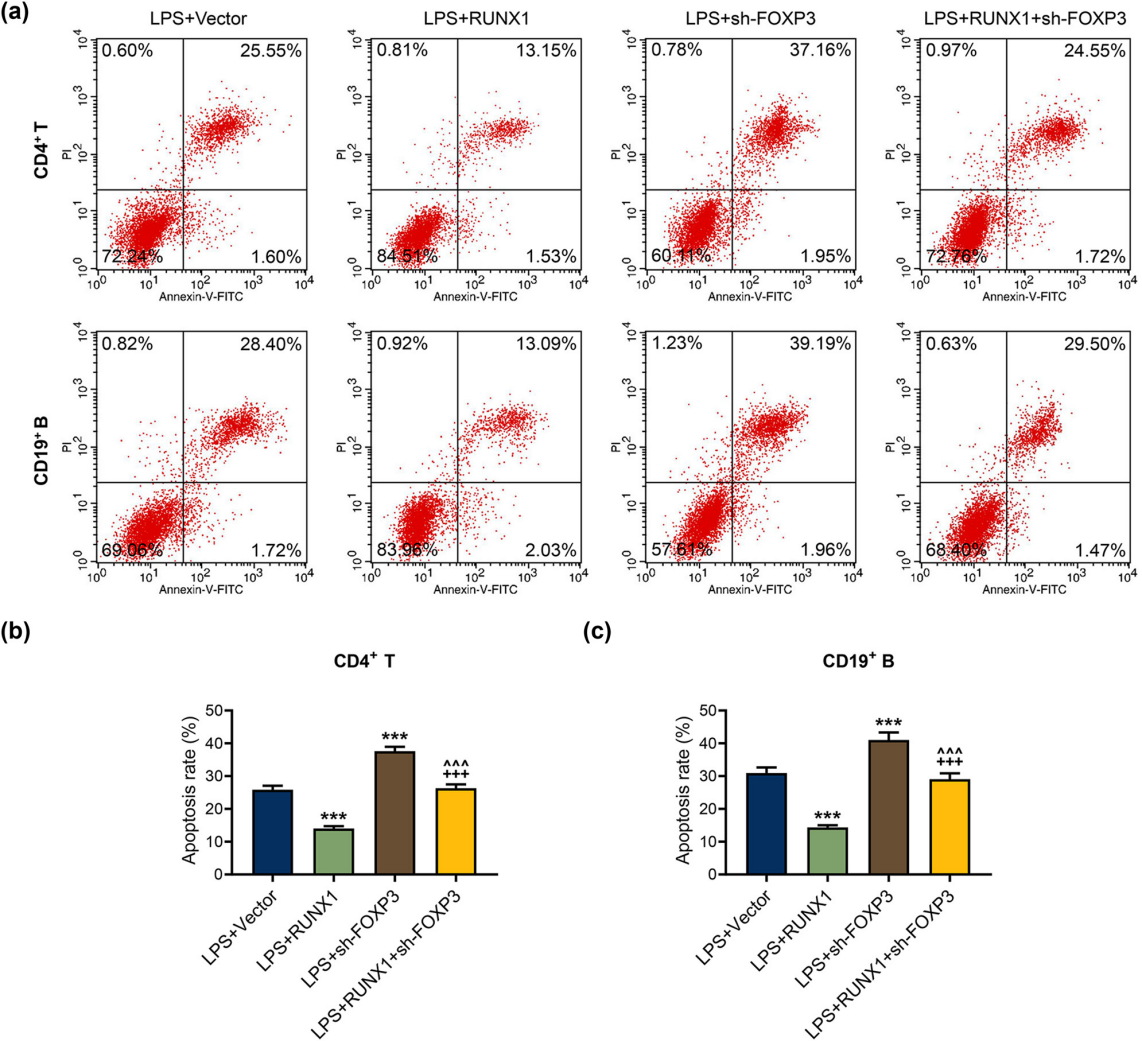
FOXP3 silencing reversed the suppression of overexpressed RUNX1 on apoptosis in LPS-mediated CD4^+^ T and CD19^+^ B cells. (a)–(c) Roles of RUNX1 and FOXP3 on apoptosis in LPS-mediated CD4^+^ T and CD19^+^ B cells were assessed by flow cytometer. ^***^ *P* < 0.001 vs LPS + Vector; ^+++^ *P* < 0.001 vs LPS + short hairpin RNA targeting FOXP3 (sh-FOXP3); ^^^^^ *P* < 0.001 vs LPS + RUNX1.

**Figure 9:**
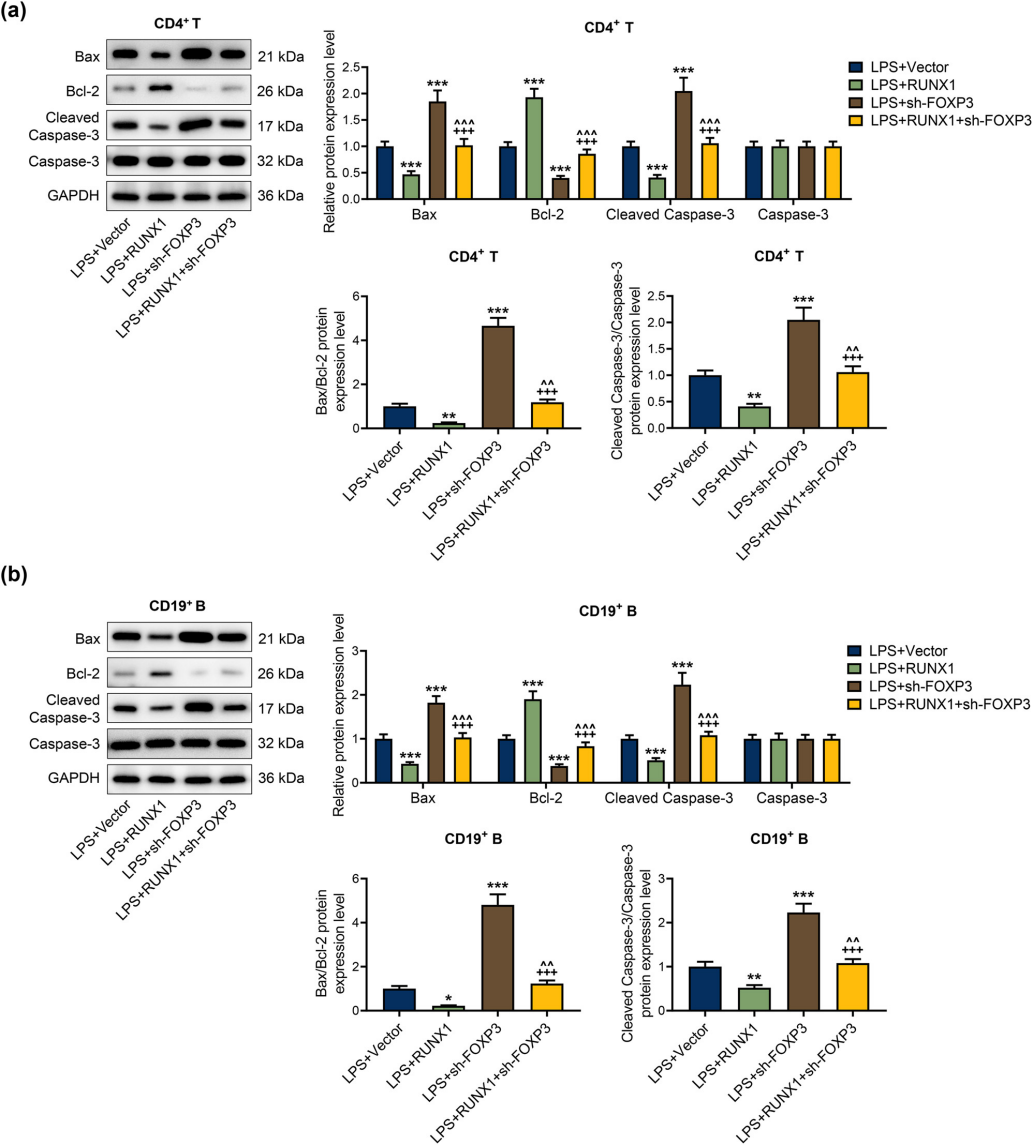
Repression of overexpressed RUNX1 on apoptosis-related factors in LPS-mediated CD4^+^ T and CD19^+^ B cells was rescued by FOXP3 silencing. (a) and (b) Roles of RUNX1 and FOXP3 on apoptosis-related factors in LPS-mediated CD4^+^ T and CD19^+^ B cells were assessed by western blot. ^*^ *P* < 0.05, ^**^ *P* < 0.01, ^***^ *P* < 0.001 vs LPS + Vector; ^+++^ *P* < 0.001 vs LPS + sh-FOXP3; ^^^^ *P* < 0.01, ^^^^^ *P* < 0.001 vs LPS + RUNX1.

In Figure 8a–c, the flow cytometer assay illustrated that FOXP3 knockdown prominently facilitated apoptosis of LPS-mediated CD4^+^ T and CD19^+^ B cells and reversed the repression of overexpressed RUNX1 on apoptosis (*P* < 0.001). Moreover, the western blot experiment illuminated that FOXP3 silencing evidently enhanced the expressions of Bax, cleaved caspase-3, the ratio of cleaved caspase-3/caspase-3, and Bax/Bcl-2, whereas weakened Bcl-2 expression in CD4^+^ T and CD19^+^ B cells mediated by LPS (Figure 9a and b, *P* < 0.01). Importantly, the modulation of overexpressed RUNX1 on apoptosis-related factors was partially offset by FOXP3 silencing (Figure 9a and b, *P* < 0.05). The data revealed the interaction of FOXP3 and RUNX1 on apoptosis of CD4^+^ T and CD19^+^ B cells in the context of LPS.

## 4 Discussion

One study has reported that RUNX1 may be a new potential therapeutic target for the prevention of sepsis [20]. In our study, we uncovered that RUNX1 and FOXP3 are under-expressed in CLP mice or LPS-mediated CD4^+^ T and CD19^+^ B cells. Importantly, RUNX1 overexpression ameliorated the survival rate, pathological damage in liver, kidney and lung tissues, decreased inflammation in serum, and apoptosis of CD4^+^ T and CD19^+^ B cells in CLP mice. Furthermore, overexpression of RUNX1 up-regulated the viability and down-regulated apoptosis in LPS-mediated CD4^+^ T and CD19^+^ B cells. Additionally, FOXP3 silencing reversed the inhibitory effect of RUNX1 on apoptosis of LPS-mediated CD4^+^ T and CD19^+^ B cells by interacting with RUNX1.

In our study, RUNX1 was decreased in CD4^+^ T and CD19^+^ B cells in CLP mice, and RUNX1 overexpression increases percent survival, and alleviates pathological damage in liver, kidney, and lung tissues of CLP mice. With the deepening of the research on the pathophysiological mechanism of sepsis, modulation of immune-inflammatory disorders has become a new direction for the treatment of sepsis [21]. In sepsis, infection first causes increased secretion of core pro-inflammatory factors such as TNF-α and IL-1β [22,23]. It has been reported that IL-6 was tightly associated with sepsis mortality and can be used as a predictor of sepsis severity due to its slow metabolism and easy detection [24,25]. In this research, our data exhibited that RUNX1 overexpression evidently attenuated inflammation in the serum of mice. These present findings indicated that RUNX1 increases survival rate of CLP mice, accompanied by decreasing pathological damage in liver, kidney, and lung tissues and serum inflammation.

Depletion of T lymphocytes is one of the significant causes of immune dysfunction, mainly manifested in the reduction of CD4^+^ T and CD8^+^ T, which further affects the immune function of the body [26]. B lymphocytes play the role of humoral immunity by specifically presenting antigens, secreting antibodies, and releasing cytokines [27]. Lymphocyte apoptosis exhibits a considerable role in immunosuppression of sepsis, and inhibition of lymphocyte apoptosis can ameliorate the prognosis of sepsis. Garofalo et al. illustrated that massive apoptosis of immune cells such as T cells, dendritic cells, and B cells were observed in many organs of deceased patients with sepsis [28]. Li et al. reported that the expressions of caspase-3, caspase-8, and caspase-9 were notably elevated, while the expressions of CD4^+/^CD8^+^ T lymphocytes and CD19^+^ B lymphocytes were evidently decreased in a mouse model of sepsis formed by CLP [29]. Reséndiz-Martínez et al. compared the children with sepsis and healthy people and found that the apoptosis of monocytes and the expression of Fas in the peripheral blood of the children with sepsis increased, and the level of apoptosis was positively correlated with the expression of Fas [30]. Moreover, another research clarified that the expressions of cytochrome C, caspase-3, caspase-8, and caspase-9 in CLP mice were obviously up-regulated, while the expression of Bcl-2 was prominently decreased, which in turn promoted T lymphocyte apoptosis [31]. Similar to the results of previous studies, we discovered a notable elevation of annexin-V binding and active caspase-3 in CD4^+^ T and CD19^+^ B cells of CLP mice; meanwhile, in *in vitro* experiments, LPS resulted in the reduction of Bcl-2 and the increase of Bax and cleaved caspase-3 in CD4^+^ T and CD19^+^ B cells. More important, we discovered that overexpressed RUNX1 repressed apoptosis of CD4^+^ T and CD19^+^ B cells in CLP mice, and in LPS-mediated CD4^+^ T and CD19^+^ B cells.

Subsequently, through literature search and bioinformatics prediction, it has been found that FOXP3 could interact with RUNX1 in T cells [32–34]. Also, FOXP3^+^ modulatory T cells were necessary for recovery from serious sepsis [16]. In addition, RUNX1 is also a gene that activates FOXP3 expression [12]. The present results clarified that RUNX1 and FOXP3 formed a complex and interacted with each other in CD4^+^ T and CD19^+^ B cells. FOXP3 forms a head-to-head dimerization, which confers distinct DNA-binding specificity and creates a docking site for RUNX1 [35]. Importantly, FOXP3 silencing reversed the suppression of overexpressed RUNX1 on apoptosis in LPS-mediated CD4^+^ T and CD19^+^ B cells. These findings indicated that RUNX1 inhibited apoptosis of LPS-mediated CD4^+^ T and CD19^+^ B cells by interacting with FOXP3.

Collectively, these findings described that the RUNX1/FOXP3 axis alleviated immunosuppression in sepsis progression by weakening T and B lymphocyte apoptosis. In future studies, we need to further examine the expression levels of apoptosis-related markers in CD4^+^ T and CD19^+^ B cells of CLP mice.

## References

[1] Rhodes A, Evans LE, Alhazzani W, Levy MM, Antonelli M, Ferrer R, et al. Surviving sepsis campaign: International Guidelines for Management of Sepsis and Septic Shock: 2016. Intensive Care Med. 2017;43(3):304–77. 10.1007/s00134-017-4683-6 28101605

[2] Xie J, Wang H, Kang Y, Zhou L, Liu Z, Qin B, et al. The epidemiology of sepsis in Chinese ICUs: a national cross-sectional survey. Crit Care Med. 2020;48(3):e209–e18. 10.1097/CCM.0000000000004155 31804299

[3] Yende S, Austin S, Rhodes A, Finfer S, Opal S, Thompson T, et al. Long-term quality of life among survivors of severe sepsis: analyses of two international trials. Crit Care Med. 2016;44(8):1461–7. 10.1097/CCM.0000000000001658 PMC4949079 26992066

[4] Goodwin AJ, Rice DA, Simpson KN, Ford DW. Frequency, cost, and risk factors of readmissions among severe sepsis survivors. Crit Care Med. 2015;43(4):738–46. 10.1097/CCM.0000000000000859 PMC4479267 25746745

[5] Venet F, Monneret G. Advances in the understanding and treatment of sepsis-induced immunosuppression. Nat Rev Nephrol. 2018;14(2):121–37. 10.1038/nrneph.2017.165 29225343

[6] Shankar-Hari M, Harrison DA, Rubenfeld GD, Rowan K. Epidemiology of sepsis and septic shock in critical care units: comparison between sepsis-2 and sepsis-3 populations using a national critical care database. Br J Anaesth. 2017;119(4):626–36. 10.1093/bja/aex234 29121281

[7] Delano MJ, Ward PA. Sepsis-induced immune dysfunction: can immune therapies reduce mortality? J Clin Invest. 2016;126(1):23–31. 10.1172/JCI82224 PMC4701539 26727230

[8] Hotchkiss RS, Tinsley KW, Swanson PE, Schmieg RE Jr., Hui JJ, Chang KC, et al. Sepsis-induced apoptosis causes progressive profound depletion of B and CD4+ T lymphocytes in humans. J Immunol. 2001;166(11):6952–63. 10.4049/jimmunol.166.11.6952 11359857

[9] Wong WF, Kohu K, Nagashima T, Funayama R, Matsumoto M, Movahed E, et al. The artificial loss of Runx1 reduces the expression of quiescence-associated transcription factors in CD4( +) T lymphocytes. Mol Immunol. 2015;68(2 Pt A):223–33. 10.1016/j.molimm.2015.08.012 26350416

[10] Hsu FC, Shapiro MJ, Dash B, Chen CC, Constans MM, Chung JY, et al. An essential role for the transcription factor runx1 in T cell maturation. Sci Rep. 2016;6:23533. 10.1038/srep23533 PMC4810436 27020276

[11] Chi Y, Huang Z, Chen Q, Xiong X, Chen K, Xu J, et al. Loss of Runx1 function results in B cell immunodeficiency but not T cell in adult zebrafish. Open Biol. 2018;8(7):180043. 10.1098/rsob.180043 PMC6070721 30045885

[12] Xu M, Liu Q, Li S, Zhang W, Huang X, Han K, et al. Increased expression of miR-338-3p impairs Treg-mediated immunosuppression in pemphigus vulgaris by targeting RUNX1. Exp Dermatol. 2020;29(7):623–9. 10.1111/exd.14111 32386260

[13] Zhang Y, Huang H, Liu W, Liu S, Wang XY, Diao ZL, et al. Endothelial progenitor cells-derived exosomal microRNA-21-5p alleviates sepsis-induced acute kidney injury by inhibiting RUNX1 expression. Cell Death Dis. 2021;12(4):335. 10.1038/s41419-021-03578-y PMC8009943 33785732

[14] Ono M. Control of regulatory T-cell differentiation and function by T-cell receptor signalling and Foxp3 transcription factor complexes. Immunology. 2020;160(1):24–37. 10.1111/imm.13178 PMC7160660 32022254

[15] Mertowska P, Mertowski S, Grywalska E, Podgajna M. The importance of the transcription factor foxp3 in the development of primary immunodeficiencies. J Clin Med. 2022;11(4):947. 10.3390/jcm11040947 PMC8874698 35207219

[16] Kühlhorn F, Rath M, Schmoeckel K, Cziupka K, Nguyen HH, Hildebrandt P, et al. Foxp3 + regulatory T cells are required for recovery from severe sepsis. PLoS One. 2013;8(5):e65109. 10.1371/journal.pone.0065109 PMC3665556 23724126

[17] Chen JX, Xu X, Zhang S. Silence of long noncoding RNA NEAT1 exerts suppressive effects on immunity during sepsis by promoting microRNA-125-dependent MCEMP1 downregulation. IUBMB Life. 2019;71(7):956–68. 10.1002/iub.2033 30883005

[18] Ren Q, Guo F, Tao S, Huang R, Ma L, Fu P. Flavonoid fisetin alleviates kidney inflammation and apoptosis via inhibiting Src-mediated NF-κB p65 and MAPK signaling pathways in septic AKI mice. Biomed Pharmacother. 2020;122:109772. 10.1016/j.biopha.2019.109772 31918290

[19] Livak KJ, Schmittgen TD. Analysis of relative gene expression data using real-time quantitative PCR and the 2(-Delta Delta C(T)) method. Methods. 2001;25(4):402–8. 10.1006/meth.2001.1262 11846609

[20] Luo MC, Zhou SY, Feng DY, Xiao J, Li WY, Xu CD, et al. Runt-related transcription factor 1 (RUNX1) binds to p50 in macrophages and enhances TLR4-triggered inflammation and septic shock. J Biol Chem. 2016;291(42):22011–20. 10.1074/jbc.M116.715953 PMC5063984 27573239

[21] Delano MJ, Ward PA. The immune system’s role in sepsis progression, resolution, and long-term outcome. Immunol Rev. 2016;274(1):330–53. 10.1111/imr.12499 PMC5111634 27782333

[22] Kurt AN, Aygun AD, Godekmerdan A, Kurt A, Dogan Y, Yilmaz E. Serum IL-1beta, IL-6, IL-8, and TNF-alpha levels in early diagnosis and management of neonatal sepsis. Mediators Inflamm. 2007;2007:31397. 10.1155/2007/31397 PMC2220039 18274637

[23] Machado JR, Soave DF, da Silva MV, de Menezes LB, Etchebehere RM, Monteiro ML, et al. Neonatal sepsis and inflammatory mediators. Mediators Inflamm. 2014;2014:269681. 10.1155/2014/269681 PMC4295603 25614712

[24] Qiu X, Zhang L, Tong Y, Qu Y, Wang H, Mu D. Interleukin-6 for early diagnosis of neonatal sepsis with premature rupture of the membranes: a meta-analysis. Medicine (Baltimore). 2018;97(47):e13146. 10.1097/MD.0000000000013146 PMC6392693 30461611

[25] Smok B, Domagalski K, Pawłowska M. Diagnostic and prognostic value of IL-6 and sTREM-1 in SIRS and sepsis in children. Mediators Inflamm. 2020;2020:8201585. 10.1155/2020/8201585 PMC7327583 32655314

[26] Pauken KE, Wherry EJ. Snapshot: T cell exhaustion. Cell. 2015;163(4):1038-.e1. 10.1016/j.cell.2015.10.054 26544946

[27] Liu Q, Lu Y, An L, Li CS. B- and T-lymphocyte attenuator expression on regulatory T-cells in patients with severe sepsis. Chin Med J (Engl). 2018;131(21):2637–9. 10.4103/0366-6999.244104 PMC6213827 30381607

[28] Garofalo AM, Lorente-Ros M, Goncalvez G, Carriedo D, Ballén-Barragán A, Villar-Fernández A, et al. Histopathological changes of organ dysfunction in sepsis. Intensive Care Med Exp. 2019;7(Suppl 1):45. 10.1186/s40635-019-0236-3 PMC6658642 31346833

[29] Li S, Zhu FX, Zhao XJ, An YZ. The immunoprotective activity of interleukin-33 in mouse model of cecal ligation and puncture-induced sepsis. Immunol Lett. 2016;169:1–7. 10.1016/j.imlet.2015.11.009 26602156

[30] Reséndiz-Martínez J, Asbun-Bojalil J, Huerta-Yepez S, Vega M. Correlation of the expression of YY1 and Fas cell surface death receptor with apoptosis of peripheral blood mononuclear cells, and the development of multiple organ dysfunction in children with sepsis. Mol Med Rep. 2017;15(5):2433–42. 10.3892/mmr.2017.6310 PMC5428261 28447715

[31] Luan YY, Yin CF, Qin QH, Dong N, Zhu XM, Sheng ZY, et al. Effect of regulatory T cells on promoting apoptosis of T lymphocyte and its regulatory mechanism in sepsis. J Interferon Cytokine Res. 2015;35(12):969–80. 10.1089/jir.2014.0235 PMC4683547 26309018

[32] Recouvreux MS, Grasso EN, Echeverria PC, Rocha-Viegas L, Castilla LH, Schere-Levy C, et al. RUNX1 and FOXP3 interplay regulates expression of breast cancer related genes. Oncotarget. 2016;7(6):6552–65. 10.18632/oncotarget.6771 PMC4872732 26735887

[33] Ono M, Yaguchi H, Ohkura N, Kitabayashi I, Nagamura Y, Nomura T, et al. Foxp3 controls regulatory T-cell function by interacting with AML1/Runx1. Nature. 2007;446(7136):685–9. 10.1038/nature05673 17377532

[34] Zhang F, Meng G, Strober W. Interactions among the transcription factors Runx1, RORgammat and Foxp3 regulate the differentiation of interleukin 17-producing T cells. Nat Immunol. 2008;9(11):1297–306. 10.1038/ni.1663 PMC4778724 18849990

[35] Leng F, Zhang W, Ramirez RN, Leon J, Zhong Y, Hou L, et al. The transcription factor FoxP3 can fold into two dimerization states with divergent implications for regulatory T cell function and immune homeostasis. Immunity. 2022;55(8):1354–69, e8. 10.1016/j.immuni.2022.07.002 PMC9907729 35926508

